# TRPM2 Channel Involvement in the Hesperidin-Mediated Potentiation of Cisplatin’s Antitumor Action in Laryngeal Carcinoma Cells

**DOI:** 10.3390/ijms27031141

**Published:** 2026-01-23

**Authors:** Ramazan Çınar, Kenan Yıldızhan, Halil İbrahim Altıner, Tarık Yağcı

**Affiliations:** 1 Department of Biophysics, Faculty of Medicine, Bilecik Seyh Edebali University, 11230 Bilecik, Türkiye; ramazan.cinar@bilecik.edu.tr; 2 Department of Biophysics, Faculty of Medicine, Van Yuzuncu Yil University, 65080 Van, Türkiye; 3 Department of Otorhinolaryngology, Faculty of Medicine, Bilecik Seyh Edebali University, 11230 Bilecik, Türkiye; halilibrahim.altiner@bilecik.edu.tr (H.İ.A.); tarik.yagci@bilecik.edu.tr (T.Y.)

**Keywords:** hesperidin, cisplatin, TRPM2 channel, oxidative stress, apoptosis

## Abstract

Cisplatin (CSP) is a first-line chemotherapeutic for laryngeal squamous cell carcinoma (LSCC), but its clinical effectiveness is limited by resistance and toxicity. Hesperidin (HESP), a citrus flavonoid, may enhance chemotherapeutic efficacy through pro-apoptotic properties. This study investigated the involvement of the transient receptor potential melastatin-2 (TRPM2) channel in the HESP-mediated potentiation of CSP-induced cytotoxicity in human laryngeal carcinoma (Hep-2) cells. Hep-2 cells were treated with CSP (25 µM), HESP (25 µM), or their combination for 24 h. The findings showed that the combined application of HESP and CSP reduced cell viability by approximately 50% (*p* < 0.001), which was the lowest compared to CSP alone. Western blot analysis revealed that TRPM2 protein expression was higher in the CSP+HESP group compared to the control group (*p* < 0.001). This synergistic treatment resulted in an increase in ROS production and a decrease in MDA levels, accompanied by a reduction in cellular GSH levels (*p* < 0.001). Furthermore, the combination therapy increased pro-inflammatory cytokines such as IL-1β and TNF-α (*p* < 0.001). Functional analyses showed that HESP treatment enhanced CSP-induced Ca^2+^ influx and altered mitochondrial membrane potential (*p* < 0.001). The pharmacological inhibition of TRPM2 with ACA and 2-APB reversed these effects, restoring redox balance and reducing cellular damage. In conclusion, HESP amplifies CSP-induced apoptosis in Hep-2 cells through TRPM2-dependent oxidative stress, Ca^2+^ dysregulation, and mitochondrial dysfunction. These findings identify TRPM2 as a mechanistic mediator of HESP-enhanced chemosensitivity in LSCC.

## 1. Introduction

Laryngeal squamous cell carcinoma (LSCC) represents one of the most prevalent and aggressive malignancies of the head and neck region, accounting for substantial morbidity and mortality worldwide. Despite advances in surgical and chemo radiotherapeutic modalities, cisplatin (CSP) remains the cornerstone of LSCC management [1,2,3]. However, the clinical efficacy of CSP is frequently compromised by the emergence of chemoresistance and by dose-limiting toxicities, including nephrotoxicity, neurotoxicity, and ototoxicity. These limitations have intensified research into combinatorial strategies that can enhance CSP’s tumoricidal potential while mitigating its adverse systemic effects. In this context, natural bioactive compounds with pleiotropic antioxidant and pro-apoptotic properties have attracted considerable attention as potential chemosensitizers in cancer therapy [4,5,6].

Hesperidin (HESP), a naturally occurring flavanone glycoside abundantly found in citrus fruits, has demonstrated diverse pharmacological activities, including anti-oxidative, anti-inflammatory, and anti-proliferative effects in various cancer models [7,8]. Recent evidence suggests that HESP can modulate multiple cellular pathways involved in oxidative stress, mitochondrial integrity, and apoptosis [9,10]. Notably, HESP has been shown to potentiate chemotherapeutic efficacy in certain malignancies by amplifying intracellular reactive oxygen species (ROS) production and promoting mitochondrial-dependent cell death [7,8,11]. Abd El Latif et al. reported that the use of HESP in combination with CSP regulated the immune response, synergistically enhanced antitumor efficacy by increasing IFN-γ and granzyme B levels, and reduced the side effects of CSP on healthy tissues [12]. Another study demonstrated that a combination of HESP and CSP exhibited synergistic cytotoxicity in osteosarcoma (U2OS) cells. This synergy was found to trigger mitochondrial-mediated apoptosis by increasing the expression of pro-apoptotic Bax and Caspase-3 genes while decreasing the levels of anti-apoptotic Bcl-2 and Survivin mRNA [13]. Karabat et al. reported that the combination of HESP and CSP synergistically induced apoptosis in human malignant melanoma (A431) cells by increasing caspase-3/7 activity and Bax expression, while also reducing cell viability [14]. Nevertheless, the molecular mechanisms underlying its potential synergism with CSP, particularly in laryngeal carcinoma, remain largely uncharacterized.

Among the molecular determinants implicated in oxidative stress-driven cell fate, the transient receptor potential melastatin-2 (TRPM2) channel has emerged as a pivotal regulator of redox homeostasis, Ca^2+^ signaling, and apoptotic responses [15,16]. TRPM2 is a non-selective Ca^2+^-permeable cation channel activated by ADP-ribose (ADPR) and oxidative stimuli such as hydrogen peroxide (H_2_O_2_). Upon activation, TRPM2 mediates sustained Ca^2+^ influx, leading to mitochondrial dysfunction, ROS amplification, and eventual cell death [17,18]. In cancer biology, TRPM2 exhibits context-dependent roles: while it can promote tumor survival by supporting bioenergetic stability, excessive or persistent activation under high oxidative stress can drive apoptotic collapse [19,20]. This dualistic function positions TRPM2 as both a survival mediator and a potential therapeutic vulnerability in malignancy.

Recent studies have identified a close interrelationship between TRPM2 activity and the efficacy of chemotherapeutic agents [21,22]. TRPM2-mediated Ca^2+^ entry has been implicated in the cellular responses to CSP, doxorubicin, and other redox-active drugs, influencing DNA damage signaling and mitochondrial permeability transition [3,23,24]. Furthermore, pharmacological modulation of TRPM2 has been shown to alter chemosensitivity in several tumour models, suggesting that channel activation or inhibition can reshape drug-induced oxidative stress and apoptosis [21,22,25]. However, whether the TRPM2 channel contributes to the HESP-mediated modulation of CSP cytotoxicity in LSCC remains unclear.

This study aimed to investigate the involvement of TRPM2 channels in HESP-mediated potentiation of the antitumor effect of CSP in Hep-2 cells. Specifically, we evaluated how HESP affects CSP-induced cytotoxicity, oxidative stress, mitochondrial dysfunction, and Ca^2+^ signalling, and whether TRPM2 inhibition modulates these effects. Through a combination of biochemical assays, fluorescence imaging, and protein expression analyses, we aimed to elucidate the mechanistic link between TRPM2 activation and HESP-induced chemosensitisation. Understanding this interaction will not only shed light on the functional duality of TRPM2 in cancer but also pave the way for novel redox-targeted combinatorial strategies to improve the therapeutic index of CSP in laryngeal carcinoma.

## 2. Results

### 2.1. HESP Potentiates the Cytotoxic Effect of CSP in Hep-2 Cells

**Figure 1:**
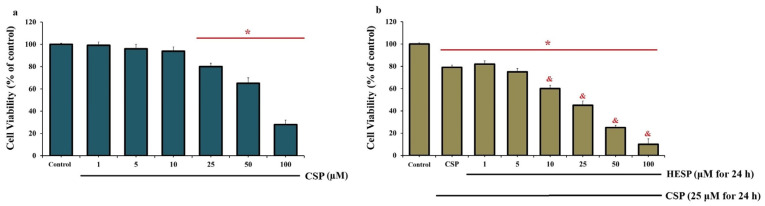
Effects of CSP and HESP on Hep-2 cell viability. (**a**) Dose-dependent cytotoxic effect of CSP (1–100 µM, 24 h) on Hep-2 cells determined by CCK-8 assay. Cell viability declined at concentrations of ≥25 µM CSP compared to the control (* *p* < 0.001). (**b**) Combined treatment of CSP (25 µM) with increasing concentrations of HESP (1–100 µM, 24 h) further reduced cell viability in a concentration-dependent manner (* *p* < 0.001 vs. control, ^&^ *p* < 0.001 vs. CSP). The combination of 25 µM CSP and HESP reduced cell viability to approximately 50%. Therefore, this dose combination was selected for subsequent experimental analyses. (Data are presented as mean ± SD, n = 6).

As shown in Figure 1, CSP exhibited a dose-dependent cytotoxic effect on Hep-2 cells, with a significant reduction in cell viability observed at concentrations of 25 µM and above following 24 h exposure (*p* < 0.001). Co-treatment with HESP further enhanced this cytotoxic response in a concentration-dependent manner. Specifically, when cells were treated with a constant submaximal dose of CSP (25 µM) and increasing concentrations of HESP (Figure 1b), cell viability progressively decreased, reaching approximately 50% at the combination of 25 µM CSP and 25 µM HESP. Based on these dose–response data, the IC_50_ value for HESP in the presence of 25 µM CSP was determined to be approximately 25 µM. Considering the dose-limiting side effects of CSP, the combination of HESP with the minimum effective CSP dose was therefore selected for subsequent analyses to evaluate the potentiating effect of HESP under submaximal CSP exposure.

### 2.2. Co-Treatment with HESP and CSP Upregulates TRPM2 Expression

**Figure 2:**
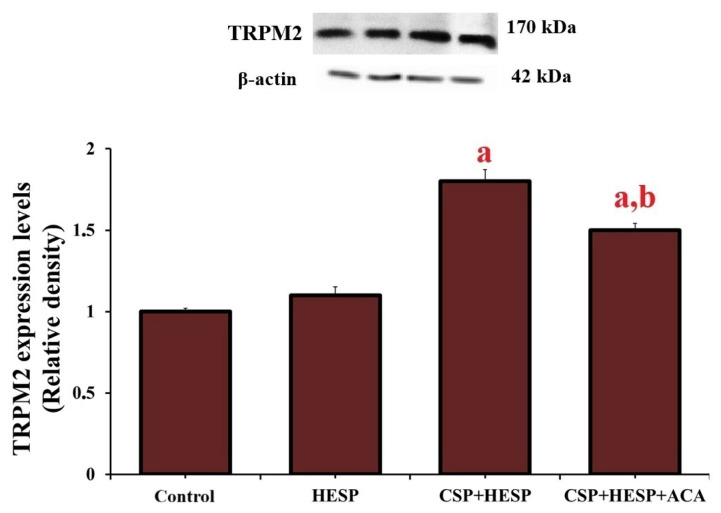
Western blot analysis of TRPM2 protein expression in Hep-2 cells. Representative immunoblots showing TRPM2 and β-actin protein bands in control, HESP (25 µM), CSP+HESP (25 µM CSP + 25 µM HESP), and CSP+HESP+ACA (25 µM, for the last 30 min of 24 h) groups for 24 h. The graph represents densitometric quantification of TRPM2 expression normalised to β-actin. Co-treatment with CSP and HESP increased TRPM2 expression compared with control and HESP alone (^a^ *p* < 0.001 vs. control and HESP groups), whereas ACA, a TRPM2 channel antagonist, attenuated this increase (^b^ *p* < 0.05 vs. CSP+HESP). (Data are expressed as mean ± SD, n = 3).

Western blot analysis showed increased TRPM2 protein expression in cells following co-exposure to CSP and HESP compared with treatments administered alone (Figure 2). Densitometric quantification revealed that TRPM2 expression was higher in the CSP+HESP group than in the control and HESP-only groups (*p* < 0.001). Importantly, the TRPM2 antagonist ACA markedly suppressed this upregulation (*p* < 0.05 vs. CSP+HESP). These findings suggest that TRPM2 activation is involved in the cellular response to combined CSP and HESP exposure.

### 2.3. TRPM2 Activation Correlates with Enhanced Oxidative and Inflammatory Responses

**Figure 3:**
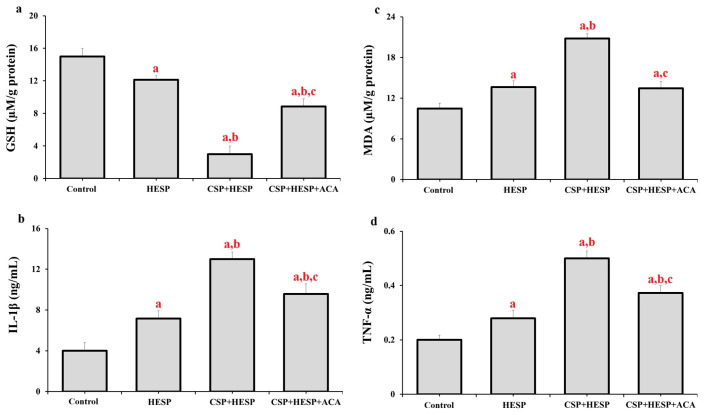
Effects of HESP, CSP, and TRPM2 inhibition (ACA) on oxidative stress and inflammatory parameters in Hep-2 cells. (**a**) GSH, (**b**) IL-1β, (**c**) MDA levels, and (**d**) TNF-α levels in the groups. (^a^ *p* < 0.001, compared to the control group; ^b^ *p* < 0.001, compared to the HESP group; ^c^ *p* < 0.001, compared to CSP+HESP group). (Data are presented as mean ± SD, n = 6).

As shown in Figure 3, co-treatment with CSP and HESP altered oxidative and inflammatory parameters. Cellular GSH levels were markedly reduced, whereas MDA, IL-1β, and TNF-α levels were elevated compared to both the control and HESP groups (*p* < 0.001). Inhibition of TRPM2 with ACA attenuated these effects, restoring GSH levels and suppressing lipid peroxidation and pro-inflammatory cytokine release (*p* < 0.001 vs. CSP+HESP). These data indicate that TRPM2 activation contributes to oxidative stress and inflammation under combined treatment conditions.

### 2.4. TRPM2 Modulates Mitochondrial Membrane Potential (ΔΨm) and ROS Generation

**Figure 4:**
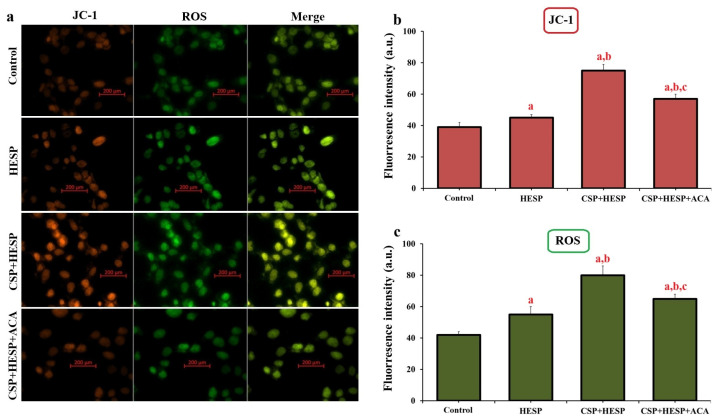
Effects of HESP, CSP, and TRPM2 inhibition on mitochondrial membrane potential and ROS generation in Hep-2 cells. (**a**) Representative fluorescence microscopy images showing JC-1 (red/orange) and ROS (green) staining in the groups. (Bar = 200 µm). (**b**) Quantitative analysis of JC-1 fluorescence intensity and (**c**) ROS fluorescence intensity (a.u.) in each group. (^a^ *p* < 0.001, compared to the control group; ^b^ *p* < 0.001, compared to the HESP group; ^c^ *p* < 0.001, compared to CSP+HESP group). (Data are presented as mean ± SD, n = 6).

Fluorescence microscopy with JC-1 and ROS probes demonstrated that co-treatment with CSP and HESP induced a profound loss of ΔΨm and excessive ROS accumulation (Figure 4a–c). Quantitative analyses confirmed depolarisation and ROS overproduction compared to control and HESP groups (*p* < 0.001). The addition of ACA mitigated both ΔΨm loss and ROS increase, implicating TRPM2-mediated Ca^2+^ influx in mitochondrial dysfunction and oxidative imbalance.

### 2.5. TRPM2-Dependent Ca^2+^ Influx in H_2_O_2_-Induced Oxidative Stress Conditions

**Figure 5:**
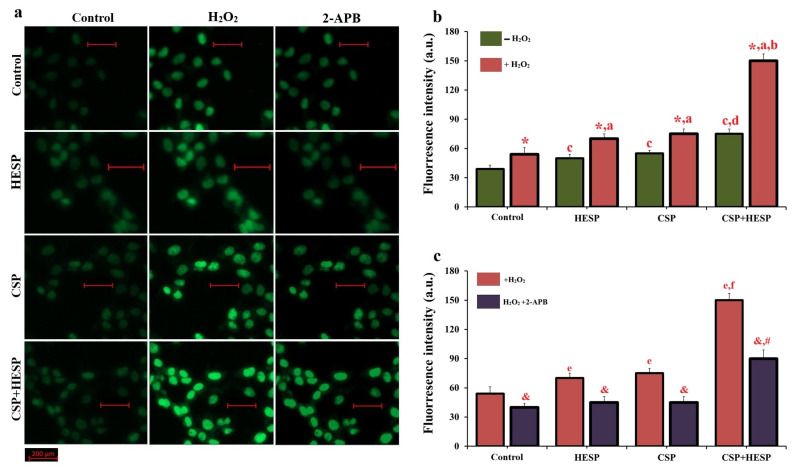
[Ca^2+^]_c_ levels and TRPM2 channel activity in Hep-2 cells under oxidative stress conditions. (**a**) Representative fluorescence microscopy images showing [Ca^2+^]_c_ fluorescence in the groups, with or without H_2_O_2_ exposure and TRPM2 inhibition by 2-APB. (Bar = 200 µm). (**b**) Effects of the TRPM2 inhibitor 2-APB (100 µM, for a period of 30 min) on H_2_O_2_-induced Hep-2 cells. 2-APB markedly suppressed [Ca^2+^]_c_ generation in both HESP and CSP groups, indicating TRPM2-dependent oxidative stress modulation. (*, ^a^ *p* < 0.01 vs. Groups with +H_2_O_2_; ^b^ *p* < 0.001 vs. CSP and HESP (Groups with +H_2_O_2_); ^c^ *p* < 0.01 vs. control group (Groups with −H_2_O)); ^d^ *p* < 0.01 vs. control, HESP and CSP (Groups with −H_2_O)). (**c**) Representative fluorescence microscopy images of Fluo-4–stained cells showing intracellular [Ca^2+^]_c_ fluorescence in control, H_2_O_2_-treated, and 2-APB-treated conditions across all experimental groups. (^e, f^ *p* < 0.01 vs. Groups with +H_2_O_2_; ^&^ *p* < 0.01 vs. Groups with +H_2_O_2_; ^#^ *p* < 0.001 vs. Groups with H_2_O_2_+2-APB). (Data are expressed as mean ± SD, n = 6).

As depicted in Figure 5, intracellular Ca^2+^ ([Ca^2+^]_c_) fluorescence intensity increased markedly in H_2_O_2_-exposed Hep-2 cells treated with CSP or HESP, supporting oxidative stress-induced TRPM2 activation. The TRPM2 inhibitor 2-APB substantially suppressed [Ca^2+^]_c_ accumulation in all groups (*p* < 0.001 vs. CSP+H_2_O_2_ and HESP+H_2_O_2_). These findings confirm that the combined treatment potentiates oxidative Ca^2+^ signalling through TRPM2, linking Ca^2+^ dysregulation to downstream apoptotic signalling events.

### 2.6. TRPM2 Activation Contributes to Cell Damage

**Figure 6:**
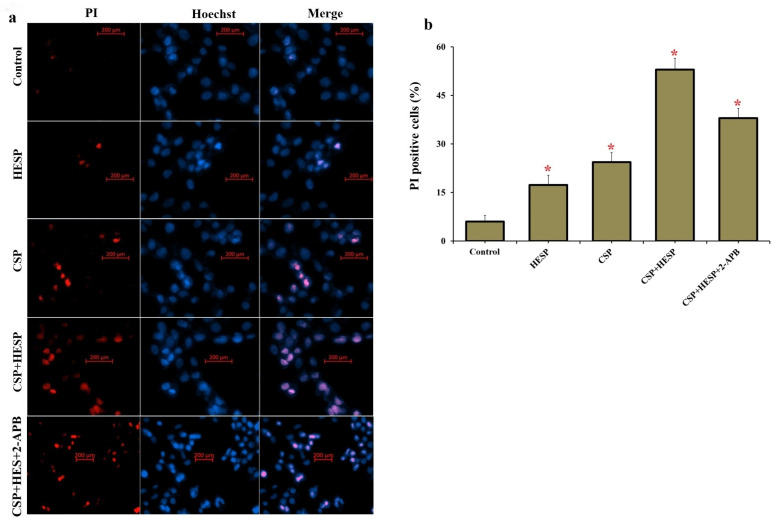
Evaluation of cell death in Hep-2 cells by PI/Hoechst dual fluorescence staining. (**a**) Representative fluorescence microscopy images showing PI-positive (red) and Hoechst-stained (blue) cells in the groups. (Bar = 200 µm). (**b**) Quantitative analysis of PI-positive cell percentage. (* *p* < 0.001). (Data are expressed as mean ± SD, n = 6).

Dual PI/Hoechst fluorescence staining (Figure 6) revealed an increase in PI-positive cells in the CSP+HESP group compared with the control and single-treatment groups (*p* < 0.001). Inhibition of TRPM2 markedly reduced PI positivity, suggesting that TRPM2 activation enhances CSP-induced apoptotic cell death. Collectively, these data demonstrate that HESP amplifies CSP cytotoxicity via TRPM2-mediated oxidative stress, Ca^2+^ influx, mitochondrial dysfunction, and apoptosis in Hep-2 cells.

## 3. Discussion

In this study, the combination of HESP and CSP markedly enhanced cytotoxicity in Hep-2 cells, and this effect was found to be tightly linked to the activation of the TRPM2 channel. Dose–response analyses demonstrated that 25 µM CSP decreased cell viability, and the addition of HESP further potentiated this decline to nearly 50%. This combination dose was selected for subsequent analyses. Mechanistically, the CSP+HESP treatment resulted in a pronounced increase in TRPM2 protein expression, accompanied by oxidative stress (decreased GSH, increased MDA, IL-1β, and TNF-α), loss of ΔΨm, and excessive ROS accumulation. The pharmacological inhibition of TRPM2 with ACA or 2-APB mitigated these changes, suppressing [Ca^2+^]_c_ increase and restoring mitochondrial integrity, implying a TRPM2-dependent regulatory mechanism. This aligns closely with recent literature identifying TRPM2 as a redox-sensitive, Ca^2+^-permeable channel that links oxidative stress to cell fate decisions. Through its gating by ADP-ribose (ADPR) and Ca^2+^, TRPM2 integrates oxidative signals with metabolic and apoptotic pathways.

The observed changes in oxidative and inflammatory biomarkers support the role of TRPM2-dependent redox imbalance in the cytotoxic effect of the CSP+HESP combination. In our study, the combined treatment increased malondialdehyde (MDA) levels, indicating increased lipid peroxidation, while decreasing intracellular glutathione (GSH), the primary non-enzymatic antioxidant responsible for maintaining redox homeostasis. Elevated MDA and depleted GSH have been widely associated with CSP-induced oxidative damage and mitochondrial dysfunction in epithelial tumor cells [26,27].

Furthermore, the rise in proinflammatory cytokines IL-1β and TNF-α indicates the activation of redox-regulated inflammatory pathways. These cytokines are known targets of NF-κB and NLRP3 inflammasome signaling, both of which are activated by ROS, and both have been previously linked to TRPM2 activation under conditions of oxidative stress [28,29]. TRPM2-mediated Ca^2+^ influx may enhance inflammatory gene transcription by increasing mitochondrial ROS production and promoting PARP1-ADPR signaling, thereby establishing a feedforward loop between oxidative stress and inflammation. Importantly, pharmacological inhibition of TRPM2 largely restored GSH levels and reduced MDA, IL-1β, and TNF-α, suggesting that the oxidative and inflammatory burden triggered by CSP+HESP is at least partially dependent on TRPM2 activity. Similarly, TRPM2-regulated modulation of oxidative stress and cytokine production has been reported in neuronal, renal, and epithelial cancer models [30,31]. Collectively, these findings highlight that TRPM2 integrates ROS generation, Ca^2+^ dysregulation, and inflammatory signalling to regulate the enhanced cytotoxic response elicited by HESP-boosted CSP treatment. The observed increase in TRPM2 expression following CSP+HESP treatment and its attenuation upon ACA or 2-APB administration suggests that TRPM2 acts not merely as a passive responder to redox imbalance, but as an active determinant of combination-induced cytotoxicity. Indeed, TRPM2 has been reported to govern diverse oncogenic processes, including DNA damage response, metabolic reprogramming, and therapy resistance [32,33].

TRPM2 is identified as a central channel that coordinates calcium signaling, oxidative stress, and energy metabolism. Its modulation within specific contexts either sustains tumor survival or accelerates apoptotic collapse [21,34]. The results indicate that under CSP and HESP exposure, excessive ROS accumulation and dissipation of the mitochondrial membrane potential occur concurrently with TRPM2 activation. In contrast, pharmacological inhibition restores cellular homeostasis, supporting a model in which TRPM2-mediated calcium influx directly contributes to mitochondrial dysfunction and subsequent cell death. Regarding the role of HESP in CSP therapy, previous studies have emphasised mainly its cytoprotective and antioxidant potential in normal tissues, particularly against CSP-induced nephrotoxicity and hepatotoxicity. TRPM2 activation facilitates sustained Ca^2+^ influx, which diffuses into mitochondria and promotes Ca^2+^-dependent ROS amplification and permeabilization, opening of TRPM2 channels. This mechanism contributes to ΔΨm collapse and apoptosis, consistent with previous findings in epithelial tumour models [35,36].

HESP exhibits context-dependent activity, serving as an antioxidant in healthy tissues but as a prooxidant in cancer cells with a high basal oxidative load. In this study, HESP acts as a prooxidant chemosensitizer by increasing CSP-induced ROS and mitochondrial dysfunction. Özdemir et al. demonstrated that HESP enhances the proapoptotic and anti-inflammatory effects of doxorubicin, potentially allowing lower chemotherapy doses and reducing systemic toxicity in cervical cancer treatment [37].

Sahu et al. reported that HESP alleviates CSP-induced oxidative injury in renal tissue by attenuating lipid peroxidation, inflammation, and apoptotic DNA fragmentation [38]. Conversely, recent cellular studies have revealed that in malignant cells, HESP can act as a pro-oxidant, promoting apoptosis through the accumulation of ROS, Ca^2+^ dysregulation, and mitochondrial depolarisation [39,40]. Jeong et al. demonstrated that HESP induces Ca^2+^-dependent mitochondrial apoptosis in prostate cancer cells, driven by increased oxidative load and caspase activation [9]. Consistent with these findings, our data reveal that in laryngeal carcinoma cells, HESP does not mitigate CSP cytotoxicity but instead amplifies it, suggesting a context and dose-dependent shift from antioxidant to pro-oxidant behaviour, particularly in metabolically stressed tumour cells.

Overall, TRPM2 is emerging as a vital mediator of HESP-CSP synergy. Recent studies in Hep-2 and other epithelial tumor lines have shown that TRPM2 plays a role in redox-induced Ca^2+^ influx, PARP1 activation, and mitochondrial permeability transition [41,42]. Inhibition of TRPM2 by ACA or 2-APB has been shown to attenuate these effects, thereby maintaining redox balance and reducing apoptotic signalling [43,44]. This study’s observation that 2-APB suppresses intracellular Ca^2+^ accumulation under H_2_O_2_ stimulation supports the hypothesis that oxidative stress activates TRPM2, which in turn facilitates Ca^2+^ induced mitochondrial activity imbalance [15,45,46]. Although TRPM2 protein expression increased after CSP+HESP treatment, functional activation was validated through H_2_O_2_-induced Ca^2+^ imaging, where TRPM2 inhibition reduced intracellular Ca^2+^ rise. Thus, the enhanced cytotoxicity is attributed primarily to TRPM2 activation rather than expression alone. This mechanistic cascade fits well with the emerging paradigm that positions TRPM2 as both a sensor and effector of redox-triggered cytotoxicity. Importantly, TRPM2 activation is closely linked to DNA damage signalling through PARP1-dependent ADPR synthesis. Under oxidative stress, PARP1 overactivation depletes NAD^+^ and produces ADPR, which opens TRPM2 channels and increases Ca^2+^ influx—creating a positive feedback loop that worsens mitochondrial and nuclear damage [47,48]. The findings of this study, which suggest that ACA/2-APB disrupts this loop, indicate a potential drug-targetable point within this pathway, aligning with existing redox-oncology models.

In light of these findings, our study highlights a promising therapeutic strategy: the use of redox-active flavonoids like HESP in combination with conventional chemotherapeutics such as CSP to reprogram tumor redox homeostasis. By specifically targeting the TRPM2 channel, this synergistic approach drives the cancer cell’s internal environment toward an irreversible collapse into apoptosis. This mechanism not only enhances the efficacy of CSP but also provides a molecular basis for overcoming chemoresistance in laryngeal carcinoma.

## 4. Materials and Methods

### 4.1. Cell Culture and Treatments

Hep-2 cells (ATCC CCL-23, Manassas, VA, USA), a human laryngeal squamous carcinoma cell line, were obtained from the Şap Institute (Ankara, Turkey). Cells were cultured in Dulbecco’s Modified Eagle Medium (DMEM; Gibco, Carlsbad, CA, USA) supplemented with 10% fetal bovine serum (FBS; Gibco), 100 U/mL penicillin, and 100 µg/mL streptomycin (Sigma-Aldrich, St. Louis, MO, USA). Cultures were maintained at 37 °C in a humidified incubator containing 5% CO_2_. To evaluate the cytotoxic and modulatory effects of CSP and HESP (Cat: A154091, Ambeed Chemical, Schleswig-Holstein, Germany), cells were treated with varying concentrations of CSP (1–100 µM) for 24 h. Based on preliminary viability data, a CSP concentration of 25 µM was selected for subsequent experiments. HESP was co-administered at concentrations ranging from 1 to 100 µM for 24 h to determine its potentiated effect. In groups involving TRPM2 modulation, the selective TRPM2 antagonists N-(p-amylcinnamoyl) anthranilic acid (ACA, 25 µM; Sigma-Aldrich) and 2-aminoethoxydiphenyl borate (2-APB, 100 µM; Sigma-Aldrich) were applied during the final 30 min of incubation. All treatments were performed in serum-free medium to prevent interference from serum antioxidants and growth factors. To ensure consistency in ELISA kits and Western blot analyses, all groups underwent a uniform 24 h treatment period. Following incubation, enzymatic and metabolic activities were halted simultaneously with either cell collection or sample freezing at −80 °C until analysis.

### 4.2. Cell Viability Assay

Cell viability was assessed using the Cell Counting Kit-8 (CCK-8; Dojindo Laboratories, Kumamoto, Japan) according to the manufacturer’s instructions. Briefly, Hep-2 cells were seeded at a density of 1 × 10^4^ cells/well in 96-well plates and treated as described above. After incubation, 10 µL of CCK-8 solution was added to each well and incubated for 2 h at 37 °C. Absorbance was measured at 450 nm using a microplate reader (BioTek Instruments, Winooski, VT, USA). Viability was expressed as a percentage relative to untreated control cells, and all measurements were performed in six independent replicates. Half-maximum inhibitory concentration (IC_50_) values were obtained from dose–response curves constructed using cell viability data obtained from the CCK-8 assay. IC_50_ values were determined by graphically interpolating the dose–response graphs to identify the concentration corresponding to approximately 50% cell viability compared to untreated control cells. Since the IC_50_ estimate was used solely to guide dose selection for subsequent analyses, no further analysis was performed.

### 4.3. Western Blot Analysis

To determine TRPM2 protein expression, whole-cell lysates were prepared using RIPA buffer (Thermo Fisher Scientific, Waltham, MA, USA) supplemented with protease and phosphatase inhibitors. Protein concentrations were quantified using the bicinchoninic acid (BCA) assay (Pierce, Rockford, IL, USA). Equal amounts of protein (30 µg per lane) were separated by SDS-polyacrylamide gel electrophoresis (10%) and transferred to PVDF membranes (Millipore, Burlington, MA, USA). Membranes were blocked with 5% non-fat dry milk in TBS-T (Tris-buffered saline, 0.1% Tween-20) for 1 h at room temperature and incubated overnight at 4 °C with the following primary antibodies: anti-TRPM2 (1:1000; Abcam, Cambridge, UK) and anti-β-actin (1:5000; Sigma-Aldrich). After washing, membranes were incubated with HRP-conjugated secondary antibodies (1:5000; Cell Signaling Technology, Danvers, MA, USA) for 1 h. Protein bands were visualised using enhanced chemiluminescence (ECL; Thermo Fisher) and quantified densitometrically with ImageJ software (version 1.54).

### 4.4. Assessment of Oxidative and Inflammatory Biomarkers

Commercial ELISA kits were used to determine the MDA, GSH, IL-1β and TNF-α levels in the collected cell supernatants. All analyses were performed according to the manufacturer’s instructions using the commercial kits (BT Lab, Wuhan, China). Absorbance values were subsequently measured using an ELISA spectrophotometer (Multiskan™ SkyHigh, Thermo Scientific, Waltham, MA, USA).

### 4.5. Measurement of Mitochondrial Membrane Potential and ROS Generation

JC-1 is a cationic carbocyanine dye that preferentially localises within mitochondria. In viable cells, JC-1 exists as a green-fluorescent monomer under depolarised mitochondrial membrane potentials, whereas under hyperpolarised conditions, it forms orange-fluorescent J-aggregates. Due to its selective mitochondrial accumulation, this dye serves as a reliable indicator of ΔΨm [49]. Changes in mΔΨ were assessed by incubating the cells with 3 µM JC-1 dye (Cat. No: T3168, Thermo Fisher Scientific, Istanbul, Türkiye). The intracellular fluorescent probe 2′,7′-dichlorofluorescein (DCF) is produced within the cytoplasm through the oxidation of its non-fluorescent precursor, dichlorofluorescein diacetate (DCFH-DA), under oxidative stress conditions [50]. Hep-2 cells were incubated with 3 µM DCFH-DA (Cat. No: C6827, Thermo Fisher Scientific). During imaging, the excitation wavelength of the argon laser was set to 488 nm, with excitation and emission wavelengths for green DCF fluorescence at 504 nm and 525 nm, respectively. After fluorescence quantification (in arbitrary units), orange and green fluorescence images corresponded to JC-1 and DCFH-DA staining, respectively.

### 4.6. Intracellular Ca^2+^ ([Ca^2+^]_c_) Imaging and TRPM2 Activity Assay

Using a Zeiss Axiovert-5 fluorescence microscope (Zeiss, Jena, Germany), the present study analyzed the increase in [Ca^2+^]_c_ concentration in Hep-2 cells induced by CSP and HESP via activation of the TRPM2 channel. After a 60 min incubation period, alterations in [Ca^2+^]_c_ were monitored through the fluorescent indicator Fluo-4 AM (1 µM; Cat. No: F1401, Thermo Fisher Scientific). The dye was excited by an argon ion laser operating at 488 nm. Despite pretreatment with 100 µM 2-APB, a known inhibitor of TRPM2 channels, exposure to 1 µM H_2_O_2_ was sufficient to elicit channel activation [12,17]. Observations were performed using a 20× magnification objective with excitation and emission wavelengths set at 494 nm and 506 nm, respectively. Fluorescence data were collected from 15 µm^2^ cytoplasmic regions and expressed as relative fluorescence intensity in arbitrary units on the green fluorescence images.

### 4.7. Evaluation of Cell Death by PI/Hoechst Dual Staining

Hep-2 cells seeded onto glass-bottom culture dishes were stained with propidium iodide (PI; 2 µg/mL, Cat. No: P1304MP, Thermo Fisher Scientific) and Hoechst 33342 (4 µM, Cat. No: 4082, Cell Signaling Technology, Danvers, MA, USA) before image acquisition using the Axiovert-5 fluorescence microscope. The obtained fluorescent micrographs were processed and quantified in ZEN Blue software (version 3.12), with PI and Hoechst visualized in the red and blue channels, respectively. Fluorescence excitation and emission settings were adjusted to 535 nm/617 nm for PI and 348 nm/455 nm for Hoechst 33342. The percentage of PI-positive cells, indicative of cell death, was determined by calculating the ratio of PI-stained cells to the total number of nuclei observed.

### 4.8. Statistical Analysis

SPSS software (version 17.0, SPSS Inc., Chicago, IL, USA) was used to analyze the data, and all data were presented as mean ± standard deviation (SD). A one-way ANOVA and Post hoc Tukey test were employed to assess all data demonstrating statistically differences between groups. Statistical significance was established at a value of *p* ≤ 0.05.

## 5. Conclusions

In summary, the combination of HESP and CSP promotes apoptosis in Hep-2 cells through a process involving TRPM2-mediated oxidative stress, calcium influx, mitochondrial dysfunction, and inflammatory activation. Pharmacological inhibition of TRPM2 (using ACA, 2-APB) reduces these effects, confirming the channel’s pivotal role in mediating cytotoxicity. These results highlight TRPM2 as both a therapeutic target and a potential biomarker for improving platinum-based chemotherapeutic strategies. Future clinical and translational research should focus on TRPM2-high head-and-neck cancer subtypes, investigating HESP+CSP TRPM2 modulation as a rational approach to overcoming CSP resistance and re-sensitising resistant tumours. This framework underscores the translational potential of combining redox-active flavonoids with conventional chemotherapeutics to reprogram tumour redox homeostasis towards irreversible apoptotic collapse.

## Data Availability

The data supporting the findings of this study are available from the corresponding author upon request.

## Abbreviations

ACA	N-(p-amylcinnamoyl) anthranilic acid
ADPR	Adenosine diphosphate ribose
ANOVA	Analysis of variance
BCA	Bicinchoninic acid
Ca^2+^	Calcium ion
CCK-8	Cell Counting Kit-8
CSP	Cisplatin
DCFH-DA	2′,7′-Dichlorofluorescein diacetate
DMEM	Dulbecco’s Modified Eagle Medium
ECL	Enhanced chemiluminescence
ELISA	Enzyme-linked immunosorbent assay
FBS	Fetal bovine serum
GSH	Glutathione
HESP	Hesperidin
H_2_O_2_	Hydrogen peroxide
IL-1β	Interleukin-1 beta
JC-1	5,5′,6,6′-Tetrachloro-1,1′,3,3′-tetraethylbenzimidazolylcarbocyanine iodide
LSCC	Laryngeal squamous cell carcinoma
MDA	Malondialdehyde
PI	Propidium iodide
PVDF	Polyvinylidene difluoride
ROS	Reactive oxygen species
SDS-PAGE	Sodium dodecyl sulfate–polyacrylamide gel electrophoresis
SD	Standard deviation
SPSS	Statistical Package for the Social Sciences
TNF-α	Tumor necrosis factor-alpha
TRPM2	Transient receptor potential melastatin-2
ΔΨm	Mitochondrial membrane potential

## References

[1] Johnson D.E., Burtness B., Leemans C.R., Lui V.W.Y., Bauman J.E., Grandis J.R. (2020). Head and neck squamous cell carcinoma. Nat. Rev. Dis. Primers.

[2] Muzaffar J., Bari S., Kirtane K., Chung C.H. (2021). Recent advances and future directions in clinical management of head and neck squamous cell carcinoma. Cancers.

[3] Yağcı T., Çınar R., Altıner H.İ., Dündar R., Yıldızhan K. (2025). The Role of TRPM2 Channel in Doxorubicin-induced Cell Damage in Laryngeal Squamous Cancer Cells. Dokl. Biochem. Biophys..

[4] Ranasinghe R., Mathai M.L., Zulli A. (2022). Cisplatin for cancer therapy and overcoming chemoresistance. Heliyon.

[5] Dasari S., Njiki S., Mbemi A., Yedjou C.G., Tchounwou P.B. (2022). Pharmacological effects of cisplatin combination with natural products in cancer chemotherapy. Int. J. Mol. Sci..

[6] Pathak K., Pathak M.P., Saikia R., Gogoi U., Sahariah J.J., Zothantluanga J.H., Samanta A., Das A. (2022). Cancer chemotherapy via natural bioactive compounds. Curr. Drug Discov. Technol..

[7] Aggarwal V., Tuli H.S., Thakral F., Singhal P., Aggarwal D., Srivastava S., Pandey A., Sak K., Varol M., Khan M.A. (2020). Molecular mechanisms of action of hesperidin in cancer: Recent trends and advancements. Exp. Biol. Med..

[8] Bayir M.H., Yıldızhan K., Altındağ F. (2023). Effect of hesperidin on sciatic nerve damage in STZ-induced diabetic neuropathy: Modulation of TRPM2 channel. Neurotox. Res..

[9] Jeong S.A., Yang C., Song J., Song G., Jeong W., Lim W. (2022). Hesperidin suppresses the proliferation of prostate cancer cells by inducing oxidative stress and disrupting Ca^2+^ homeostasis. Antioxidants.

[10] Yıldızhan K., Bayir M.H., Huyut Z., Altındağ F. (2025). Effect of Hesperidin on Lipid Profile, Inflammation and Apoptosis in Experimental Diabetes. Dokl. Biochem. Biophys..

[11] Pandey P., Khan F. (2021). A mechanistic review of the anticancer potential of hesperidin, a natural flavonoid from citrus fruits. Nutr. Res..

[12] Abd El Latif H.M., El-Morsy A.M., Ibrahim H.M., Morsi D.S. (2024). Synergistic Antineoplastic and Immunomodulatory Effects of Hesperidin in Ehrlich Ascites Carcinoma Tumor Model Treated with Cisplatin. Jordan J. Biol. Sci..

[13] Ziyadanoğulları M.O., Tuncer M.C., Özdemir İ. (2025). Antitumor Effects of Hesperidin and Cisplatin on Human Osteosarcoma Cells Through Inhibiting Proliferation and Inducing Mitochondrial-Mediated Apoptosis. Medicina.

[14] Karabat M.U., Tuncer M.C., Özdemir İ. (2025). In Vitro Evaluation of Cytotoxic and Pro-Apoptotic Effects of Hesperidin Alone and in Combination with Cisplatin on Human Malignant Melanoma Cell Line (A431). Pharmaceuticals.

[15] Yıldızhan K., Nazıroğlu M. (2023). NMDA receptor activation stimulates hypoxia-induced TRPM2 channel activation, mitochondrial oxidative stress, and apoptosis in neuronal cell line: Modular role of memantine. Brain Res..

[16] Bao L., Festa F., Freet C.S., Lee J.P., Hirschler-Laszkiewicz I.M., Chen S.-J., Keefer K.A., Wang H.-G., Patterson A.D., Cheung J.Y. (2019). The human transient receptor potential melastatin 2 ion channel modulates ROS through Nrf2. Sci. Rep..

[17] Nazıroğlu M. (2007). New molecular mechanisms on the activation of TRPM2 channels by oxidative stress and ADP-ribose. Neurochem. Res..

[18] Nazıroğlu M. (2011). TRPM2 cation channels, oxidative stress and neurological diseases: Where are we now?. Neurochem. Res..

[19] Malko P., Ding R., Jiang L.-H. (2021). TRPM2 channel in oxidative stress-induced mitochondrial dysfunction and apoptotic cell death. Adv. Protein Chem. Struct. Biol..

[20] Osmanlıoğlu H.Ö., Yıldırım M.K., Akyuva Y., Yıldızhan K., Nazıroğlu M. (2020). Morphine induces apoptosis, inflammation, and mitochondrial oxidative stress via activation of TRPM2 channel and nitric oxide signaling pathways in the hippocampus. Mol. Neurobiol..

[21] Ali E.S., Chakrabarty B., Ramproshad S., Mondal B., Kundu N., Sarkar C., Sharifi-Rad J., Calina D., Cho W.C. (2023). TRPM2-mediated Ca^2+^ signaling as a potential therapeutic target in cancer treatment: An updated review of its role in survival and proliferation of cancer cells. Cell Commun. Signal..

[22] Ji D., Luo Z.-W., Ovcjak A., Alanazi R., Bao M.-H., Feng Z.-P., Sun H.-S. (2022). Role of TRPM2 in brain tumours and potential as a drug target. Acta Pharmacol. Sin..

[23] Shitaw E.E., AlAhmad M., Sivaprasadarao A. (2025). Inter-Organelle Crosstalk in Oxidative Distress: A Unified TRPM2-NOX2 Mediated Vicious Cycle Involving Ca^2+^, Zn^2+^, and ROS Amplification. Antioxidants.

[24] Yu B., Jin L., Yao X., Zhang Y., Zhang G., Wang F., Su X., Fang Q., Xiao L., Yang Y. (2023). TRPM2 protects against cisplatin-induced acute kidney injury and mitochondrial dysfunction via modulating autophagy. Theranostics.

[25] Singh R., Adhya P., Sharma S.S. (2021). Redox-sensitive TRP channels: A promising pharmacological target in chemotherapy-induced peripheral neuropathy. Expert Opin. Ther. Targets.

[26] Santos N., Catão C., Martins N., Curti C., Bianchi M.d.L.P., Santos A.C.d. (2007). Cisplatin-induced nephrotoxicity is associated with oxidative stress, redox state unbalance, impairment of energetic metabolism and apoptosis in rat kidney mitochondria. Arch. Toxicol..

[27] Qian W., Nishikawa M., Haque A.M., Hirose M., Mashimo M., Sato E., Inoue M. (2005). Mitochondrial density determines the cellular sensitivity to cisplatin-induced cell death. Am. J. Physiol.-Cell Physiol..

[28] Zhong Z., Zhai Y., Liang S., Mori Y., Han R., Sutterwala F.S., Qiao L. (2013). TRPM2 links oxidative stress to NLRP3 inflammasome activation. Nat. Commun..

[29] Wang L., Negro R., Wu H. (2020). TRPM2, linking oxidative stress and Ca^2+^ permeation to NLRP3 inflammasome activation. Curr. Opin. Immunol..

[30] Miller B.A. (2019). TRPM2 in Cancer. Cell Calcium.

[31] Chen Z., Wu Z., Xu T., Yu F., Wang D. (2025). TRPM2: A pivotal player in tumor progression and a promising therapeutic target. Cancer Cell Int..

[32] Giannaccari M., Florindi C., Bloise N., Moccia F., Lodola F., Visai L. (2025). TRP channels and cancer modulation: A voyage beyond metabolic reprogramming, oxidative stress and the advent of nanotechnologies in targeted therapy. J. Exp. Clin. Cancer Res..

[33] Ciaglia T., Vestuto V., Bertamino A., González-Muñiz R., Gómez-Monterrey I. (2023). On the modulation of TRPM channels: Current perspectives and anticancer therapeutic implications. Front. Oncol..

[34] Piciu F., Balas M., Badea M.A., Cucu D. (2023). TRP channels in tumoral processes mediated by oxidative stress and inflammation. Antioxidants.

[35] Aboraya D.M., El Baz A., Risha E.F., Abdelhamid F.M. (2022). Hesperidin ameliorates cisplatin induced hepatotoxicity and attenuates oxidative damage, cell apoptosis, and inflammation in rats. Saudi J. Biol. Sci..

[36] Zhou J., Nie R.-C., Yin Y.-X., Cai X.-X., Xie D., Cai M.-Y. (2022). Protective effect of natural antioxidants on reducing Cisplatin-Induced nephrotoxicity. Dis. Markers.

[37] Özdemir İ., Afşin Y., Tuncer M.C., Öztürk Ş. (2025). Combined hesperidin and doxorubicin treatment induces apoptosis and modulates inflammatory cytokines in HeLa cervical cancer cells. Int. J. Mol. Sci..

[38] Sahu B.D., Kuncha M., Sindhura G.J., Sistla R. (2013). Hesperidin attenuates cisplatin-induced acute renal injury by decreasing oxidative stress, inflammation and DNA damage. Phytomedicine.

[39] Zhang Q., Yang Z., Ou X., Zhang M., Ji R., Wu G. (2025). Hesperidin Inhibits Oxidative Stress and Apoptosis of Granulosa Cells in Polycystic Ovarian Syndrome Through the JAK2/STAT3 and PI3K/AKT Pathways. Phytother. Res..

[40] Yumnam S., Hong G.E., Raha S., Saralamma V.V.G., Lee H.J., Lee W.S., Kim E.H., Kim G.S. (2016). Mitochondrial dysfunction and Ca^2+^ overload contributes to hesperidin induced paraptosis in hepatoblastoma cells, HepG2. J. Cell. Physiol..

[41] Yazgan Y., Cinar R. (2025). Gallic Acid Enhances Cisplatin-induced Death of Human Laryngeal Cancer Cells by Activating the TRPM2 Channel. Dokl. Biochem. Biophys..

[42] Kumbul Y.Ç., Nazıroğlu M. (2022). Paclitaxel promotes oxidative stress–mediated human laryngeal squamous tumor cell death through the stimulation of calcium and zinc signaling pathways: No synergic action of melatonin. Biol. Trace Elem. Res..

[43] Zhang H., Yu P., Lin H., Jin Z., Zhao S., Zhang Y., Xu Q., Jin H., Liu Z., Yang W. (2021). The discovery of novel ACA derivatives as specific TRPM2 inhibitors that reduce ischemic injury both in vitro and in vivo. J. Med. Chem..

[44] Malko P., Syed Mortadza S.A., McWilliam J., Jiang L.H. (2019). TRPM2 Channel in Microglia as a New Player in Neuroinflammation Associated with a Spectrum of Central Nervous System Pathologies. Front. Pharmacol..

[45] Huang P., Qu C., Rao Z., Wu D., Zhao J. (2024). Bidirectional regulation mechanism of TRPM2 channel: Role in oxidative stress, inflammation and ischemia-reperfusion injury. Front. Immunol..

[46] Yazğan Y., Yıldızhan K. (2025). Chrysin Boosts the Cell Death and Anticancer Actions of Doxorubicin by Stimulating the TRPM2 Channel in Glioblastoma Cells. Mol. Biol..

[47] Sharif T., Martell E., Dai C., Ghassemi-Rad M.S., Kennedy B.E., Lee P.W., Gujar S. (2019). Regulation of cancer and cancer-related genes via NAD+. Antioxid. Redox Signal..

[48] Buelow B., Song Y., Scharenberg A.M. (2008). The Poly (ADP-ribose) polymerase PARP-1 is required for oxidative stress-induced TRPM2 activation in lymphocytes. J. Biol. Chem..

[49] Carrageta D.F., Freire-Brito L., Oliveira P.F., Alves M.G. (2022). Evaluation of human spermatozoa mitochondrial membrane potential using the JC-1 Dye. Curr. Protoc..

[50] Çınar R., Yıldızhan K. (2025). Curcumin protects against MPP+-induced neurotoxicity in SH-SY5Y cells by modulating the TRPV4 channel. Mol. Biol. Rep..

